# An Alarming Change: What Caused an Instance of Inappropriate Detection in a Wearable Cardioverter-defibrillator?

**DOI:** 10.19102/icrm.2020.110201

**Published:** 2020-02-15

**Authors:** Kardie Tobb, Raffaele Corbisiero, Frank Fish, Pedram Kazemian, David Muller

**Affiliations:** ^1^Deborah Heart & Lung Center, Browns Mills, NJ, USA; ^2^Abbott Laboratories, Sylmar, CA, USA

**Keywords:** Cardiac resynchronization therapy, heart failure, wearable cardioverter-defibrillator

## Abstract

We describe the case of a patient who received cardiac resynchronization pacemaker therapy (CRT-P) incorporating bridge protection via a wearable cardioverter-defibrillator (WCD) while awaiting final evaluation for permanent placement of an implantable CRT device with defibrillation capabilities. In an attempt to mitigate symptoms of heart failure, reprogramming of the CRT-P with a V–V offset caused an unusual interaction and alarm from the WCD. This case highlights a unique interaction not previously reported and is clinically relevant to patients awaiting final evaluation for implantable defibrillator system placement.

## Introduction

The Zoll LifeVest^®^ (Zoll Medical, Chelmsford, MA, USA) wearable cardioverter-defibrillator (WCD) is a wearable external cardioverter-defibrillator that may be considered for use in patients who are at high risk of sudden arrhythmic death and who require protection but who have yet to meet the strict criteria for permanent implantable device placement. Clinical evidence supports the use of these devices in such situations.^1–4^ This device has been designed as a microprocessor-based and programmable patient-worn defibrillator that electrocardiographically monitors the patient’s heart function and automatically delivers electrical therapy to treat syncopal ventricular tachyarrhythmias or sudden cardiac arrest.

Arrhythmia detection is performed by WCDs using four electrodes placed circularly in the chest garment. Importantly, this unipolar-like detection approach might increase the risk of electrical interference of other electrical devices, which could prompt inappropriate alarms or shock delivery in patients with WCDs.

This case report discusses an unusual interaction wherein a cardiac resynchronization therapy pacemaker (CRT-P) pacing output interfered with the WCD detection algorithm, resulting in an alarm.

## Case presentation

**Figure 1:**
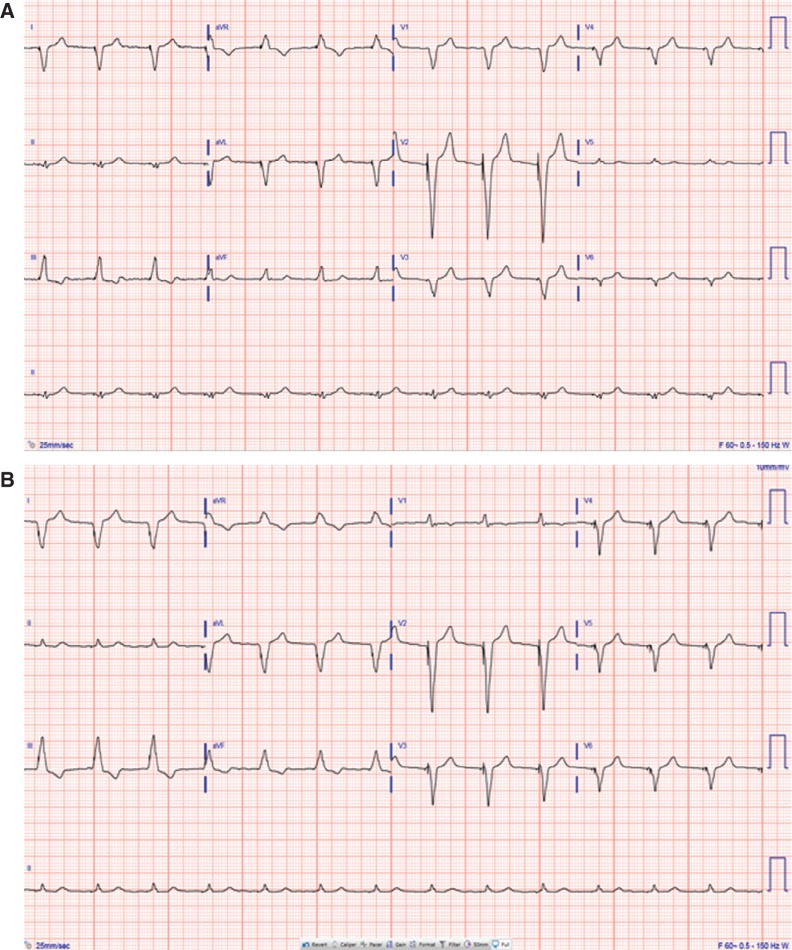
**A:** Presenting ECG with precordial leads showing a left bundle branch block pattern with no initial R-wave in V1 and a QRS duration of 148 ms. The CRT-P device was programmed with the LV–RV offset programmed to the simultaneous mode. **B:** Restoration of an initial R-wave in V1 and a QRS duration of 140 ms with the CRT-P device programmed with an LV–RV offset of −40 LV.

A 76-year-old male with a past medical history of atrial fibrillation on Eliquis^®^ (Bristol-Myers Squibb, New York, NY, USA), who had previously received a CRT-P device, presented with cardiomyopathy with an ejection fraction (EF) of 18% according to a multigated acquisition scan. Upon presentation, the patient was wearing a LifeVest^®^ WCD (Zoll Medical, Chelmsford, MA, USA) and requested a second opinion in our ambulatory care clinic regarding the recommendation for an upgrade from his current CRT-P system to a CRT device with defibrillation capabilities (CRT-D) due to his diminished EF in addition to heart failure symptoms. Coincidentally, the patient was scheduled on the same day at another facility for an implant procedure. A transthoracic echocardiogram showed moderate mitral insufficiency, aortic stenosis (with a dimensionless index of 0.33), and a revised EF result of 26%. His other comorbidities included hypertension, sleep apnea, and hyperlipidemia. An electrocardiogram (ECG) recorded during the visit showed evidence for a CRT-P; however, the precordial leads demonstrated a left bundle branch block pattern with no initial R-wave in V1, consistent with suboptimal biventricular pacing^5^ **(Figure 1)**. As a result of this ECG, the patient’s device was interrogated, which showed well-functioning leads with multiple episodes of nonsustained ventricular tachycardia and programming of the V–V offset set to a simultaneous mode. It was felt that the patient might exhibit a better response if he were to undergo electrocardiographic V–V optimization, resulting in restoration of an initial R-wave in V1 by programming a left ventricle–right ventricle (LV–RV) offset of 40 ms LV-first. The subsequent ECG demonstrated not only a more prominent R-wave in lead V1 but also some narrowing of the QRS duration, both of which are more favorable to reverse remodeling in CRT patients.^5^ The patient’s exercise capacity during the six-minute walk test as well as his heart failure symptoms improved after changes were made. After reviewing all relevant clinical information and discussing the risks and benefits of the procedure with the patient, a mutual decision to proceed with upgrade to CRT-D was made. In the interim, the patient was directed to continue wearing the WCD for protection from sudden cardiac death.

After returning home, the patient called within one hour to report repeated alarms from his WCD. He was reassured and advised to notify the WCD representative. However, a few hours later, the alarms remained persistent, with no shocks delivered. The patient presented again at our institution’s device clinic, during which time, it was determined that the patient’s WCD was inappropriately counting his ventricular rate due to the newly changed LV–RV offset. At the conclusion of the visit, adjustments were made to the device, such as reprogramming the LV–RV offset back to simultaneous mode, which seemed to subsequently solve the problem. The patient has since undergone successful CRT-D implantation.

## Discussion

The LifeVest^®^ WCD (Zoll Medical, Chelmsford, MA, USA) utilizes the TruVector™ detection approach to monitor signals from the wearable electrode belt and functions by employing a combination of heart rate and morphology analysis based on the baseline template of the patient’s morphology. This algorithm uses four electrodes embedded on the belt to assess the wearer’s heart rate. In this way, the ECG patterns caused by physiological arrhythmias are discriminated from patterns caused by nonphysiological signals. The detection algorithm includes a variety of features to identify and remove interference, reduce noise, and evaluate the quality of electrode signals.

In our case, it was originally hypothesized that the change in morphology caused a nonmatch and that a new template was required. Furthermore, due to double counting of the optimized ventricular electrogram, the rate criteria for ventricular tachycardia detection was met even at physiologic rates. The combination of unmatched morphology and meeting of rate criteria for ventricular tachycardia detection due to double counting resulted in WCD arrhythmia alerts, which were overcome by reprogramming the device to simultaneous RV–LV pacing.

This case represents the importance of two different considerations to maintain in patients with combined CRT devices and WCDs. The patient remained protected while considering the need for defibrillator implantation. However, careful evaluation should be done when programming CRT devices with V–V offsets in patients wearing WCDs. Changes in V–V offsets to values of greater than 5 ms or the introduction of any programming that may alter the device’s ability to detect, diagnose, and deliver appropriate therapy should be done cautiously. Although no therapies were delivered in this case, the programming changes that alleviated the patient’s symptoms with activity could not be programmed due to the WCD detection and alarms.
